# Deep Phenotyping of T-Cells Derived From the Aneurysm Wall in a Pediatric Case of Subarachnoid Hemorrhage

**DOI:** 10.3389/fimmu.2022.866558

**Published:** 2022-05-31

**Authors:** Giorgia Moschetti, Chiara Vasco, Francesca Clemente, Eugenia Galeota, Marco Carbonara, Mauro Pluderi, Marco Locatelli, Nino Stocchetti, Sergio Abrignani, Elisa R Zanier, Fabrizio Ortolano, Tommaso Zoerle, Jens Geginat

**Affiliations:** ^1^National Institute for Molecular Genetics (INGM), Milan, Italy; ^2^Neuroscience Intensive Care Unit, Department of Anesthesia and Critical Care, Fondazione IRCCS Cà Granda Ospedale Maggiore Policlinico, Milan, Italy; ^3^Department of Neurosurgery, Fondazione IRCCS Ca’ Granda Ospedale Maggiore Policlinico, Milan, Italy; ^4^Department of Pathophysiology and Transplantation, University of Milan, Milan, Italy; ^5^Department of Clinical Sciences and Community Health University Milan, Milan, Italy; ^6^Laboratory of Acute Brain Injury and Therapeutic Strategies, Department of Neuroscience, Mario Negri Institute for Pharmacological Research IRCCS, Milan, Italy

**Keywords:** intracranial aneurysm, subarachnoid haemorrhage, T-cells, flow cytometry, phenotype

## Abstract

Intracranial aneurysms (IAs) are very rare in children, and the characteristics of the T-cells in the IA wall are largely unknown. A comatose 7-years-old child was admitted to our center because of a subarachnoid hemorrhage due to a ruptured giant aneurysm of the right middle cerebral artery. Two days after the aneurysm clipping the patient was fully awake with left hemiparesis. T-cells from the IA wall and from peripheral blood of this patient were analyzed by multi-dimensional flow cytometry. Unbiased analysis, based on the use of FlowSOM clustering and dimensionality reduction technique UMAP, indicated that there was virtually no overlap between circulating and tissue-infiltrating T-cells. Thus, naïve T-cells and canonical memory T-cells were largely restricted to peripheral blood, while CD4^-^CD8^-^T-cells were strongly enriched in the IA wall. The unique CD4^+^, CD8^+^ and CD4^-^CD8^-^T-cell clusters from the IA wall expressed high levels of CCR5, Granzyme B and CD69, displaying thus characteristics of cytotoxic and tissue-resident effector cells. Low Ki67 expression indicated that they were nevertheless in a resting state. Among regulatory T-cell subsets, Eomes^+^Tr1-like cells were strongly enriched in the IA wall. Finally, analysis of cytokine producing capacities unveiled that the IA wall contained poly-functional T-cells, which expressed predominantly IFN-γ, TNF and IL-2. CD4^+^T-cells co-expressed also CD40L, and produced some IL-17, GM-CSF and IL-10. This report provides to our knowledge the first detailed characterization of the human T-cell compartment in the IA wall.

## Introduction

An intracranial aneurysm (IA) is a focal dilatation of a cerebral vessel (1). IAs are very rare in children but they cause more than 10% of hemorrhagic strokes in the pediatric population (2) with high mortality and morbidity (3, 4). The pathogenesis and the natural history of these pediatric vascular malformations are largely unknown with 50% of cases presenting without risk factors or underlying diseases (e.g. head trauma, infections, tumors, inflammatory diseases, excessive hemodynamic stress) (5, 6). Data from adult patients and animal experiments suggested that endothelial damage and dysfunction, vascular smooth muscle cell modulation and extracellular matrix remodeling are the main steps, leading to arterial wall degeneration and to IA formation (7–10) Different pro-inflammatory mediators seem to be crucial in these complex processes, including cytokines like IL-1β, IL-6 and TNF-α, matrix metalloproteinases (MMP), monocyte chemoattractant protein 1 (MCP1), reactive oxygen species, complement and several growth factors. In addition, immune cells were identified in the aneurysm wall of human subjects, mainly macrophages and T-cells (11, 12). However, while macrophages are considered to be fundamental for IA formation, progression and rupture (13), the role of T-cells is less clear. T-cells play a key role in orchestrating inflammation, with different sub-populations involved in the production of pro- or anti-inflammatory cytokines and in the modulation of the activity of other immune cells (14). However, the contribution of these sub-populations in the IA pathogenesis and rupture is largely unexplored, limiting our understanding of these complex processes that could represent a new therapeutic target for intracranial bleeding prevention.

The aim of this study was thus to characterize in depth, for the first time, T-cells that infiltrate the aneurysm wall, derived from a pediatric idiopathic IA.

## Material and Methods

### Cell Isolation and Purification

A blood sample and a piece of the IA wall were collected during surgery. As a control, peripheral blood from a sex-matched 4 years-old child was obtained. Written informed consent was signed by both parents for further analysis and data publication. The study was approved by the Ethics Committee of the Fondazione IRCCS Ca’ Granda–Ospedale Maggiore Policlinico (3.11/2021-680). Peripheral blood mononuclear cells (PBMC) were isolated by density gradient centrifugation by Ficoll density gradient (Amersham Pharmacia Biotech, Uppsala, Sweden). Cells from the aneurysm wall were collected using a Tumor dissociation kit (Miltenyi Biotec, Bergisch Gladbach; Germany) according to the manufacturer’s instructions. After dissociation, the sample was filtered (70µm) to remove any remaining larger particles from the single-cell suspension and lymphocytes were isolated by Ficoll density gradient.

### Flow Cytometry

T-cell subsets were analysed for the expression of surface markers, transcription factors and cytokines by staining with various combinations of fluorochrome-conjugated monoclonal antibodies (**Supplementary Table 1**). For intracellular cytokine detection, T cells were incubated for 4 hours in the presence or absence of phorbol 12-myristate 13- acetate (PMA), ionomycin in complete RPMI (10% FBS, 1 mmol/L sodium pyruvate, 10 mmol/L nonessential amino acids, and 1% penicillin/streptomycin), and with BrefeldinA (Sigma, St Louis, Mo) for an additional 2 hours. After fixation with Intracellular Fixation & Permeabilization Buffer Set (Thermo Fisher Scientific, Waltham, Massachusetts) cells were permeabilized with Permeabilization Buffer (BD Biosciences). Analysis was performed with a FACSSymphony™ cytometer (Becton Dickinson, Franklin Lakes, NJ) and analysed using FlowJo software (BD Biosciences).

### Bioinformatic Analysis

Flow cytometry data were imported into FlowJo software (version 10.8.0) to compensate fluorescence spreading; dead cells and debris were excluded from the analysis and CD3+ T cell population was selected for further analysis. A random down-sample to 5000 events in the CD3+ T-cells compartment was exported as FCS file for further analysis in R software (version 4.0.2).

Sample batches were read using read.flowSet (2.6.0) from the flowCore R package. We applied the Logicle transformation that allows the use of multiple samples to estimate transformation parameters. To reduce batch effect due to technical and not to biological variation we normalized the signal of each marker with the function gaussNorm from the flowStat package (4.6.0). After the batch-specific pre-processing, samples were concatenated into a SingleCellExperiment object in R using the function *prepData* from the CATALYST R package (15). Dimensionality reduction by UMAP was subsequently applied to visualize relative proximities of cells within reduced dimensions. Two parallel analyses were applied for each of the two panels. We performed high-resolution, unsupervised clustering and meta-clustering using FlowSOM (2.2.0) and ConsensusClusterPlus (1.58.0) packages following the workflow in Nowicka et al. (15). The T cell compartment was clustered based on the expression of 15 markers for Surface panel (CD4, CD8, CCR7, CD45RA, CD27, CD25, CD127, EOMES, GRANZYME_K, GRANZYME_B, CCR6, CXCR3, CCR5, CD69, 41BB) and 15 markers for Cytokines panel (CD4, CD8, CD127, FOXP3, EOMES, GRANZYME_K, GRANZYME_B, CCR6, CXCR3, T_BET, IL2, GM_CSF, TNFA, IFNG, CD40L). We excluded from the analysis markers that identified only very low numbers of positive cells, i.e. IFN-γ, FOXP3, KI67, and CD40L from the surface panel for unstimulated cells and KI67, IL10, IL17A and IL17F for the cytokine panel of PMA and Ionomycin-stimulated cells. Manually annotated clusters were subsequently visualized on the UMAP. Functional pseudo-time analysis to infer the differentiation trajectory of cells was carried out by DiffusionMaps (15) using the function *runDiffusionMap* on the *SingleCellExperiment* object using default parameters.

## Results

### A Pediatric Case of Intracranial Aneurysm

**Figure 1:**
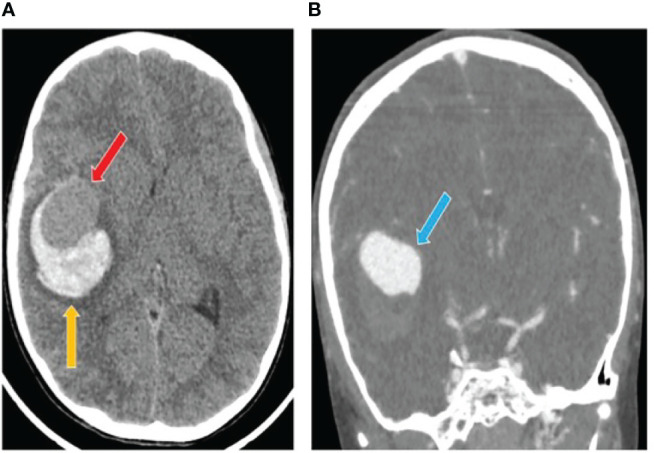
Computed tomography (CT) scan after intracranial aneurysm rupture. **(A)** The initial head CT showed a thin subarachnoid hemorrhage and a large intra-parenchymal hemorrhage (yellow arrow) including an iso-dense region (red arrow). **(B)** The angio-TC showed a cerebral aneurysm originating from the right middle cerebral artery (blue arrow).

A previously healthy 7-years-old male child had a sudden onset headache followed by loss of consciousness while he was playing at home. He was intubated on the scene and transferred to the emergency department. The head computed tomography scan (CT) showed a thin subarachnoid hemorrhage (SAH), a large intra-parenchymal hemorrhage (ICH) and mid-line shift. A giant aneurysm of the right middle cerebral artery was identified by angio-CT (**Figure 1**). The patient underwent craniotomy for ICH evacuation and intracranial aneurysm (IA) clipping. He was admitted to the Neuro Intensive Care Unit and required standard post-operative care with sedation, artificial ventilation and hemodynamic support. Two days later he was fully awake with left hemiparesis and was extubated. At 9-months follow-up, the parents reported resumption of normal life without significant disabilities despite a mild left hemiparesis.

### Unbiased Analysis of T-Cell Phenotypes Unveiled Virtually No Overlap Between T-Cell Subsets From the Blood and the Aneurysm Wall

**Figure 2:**
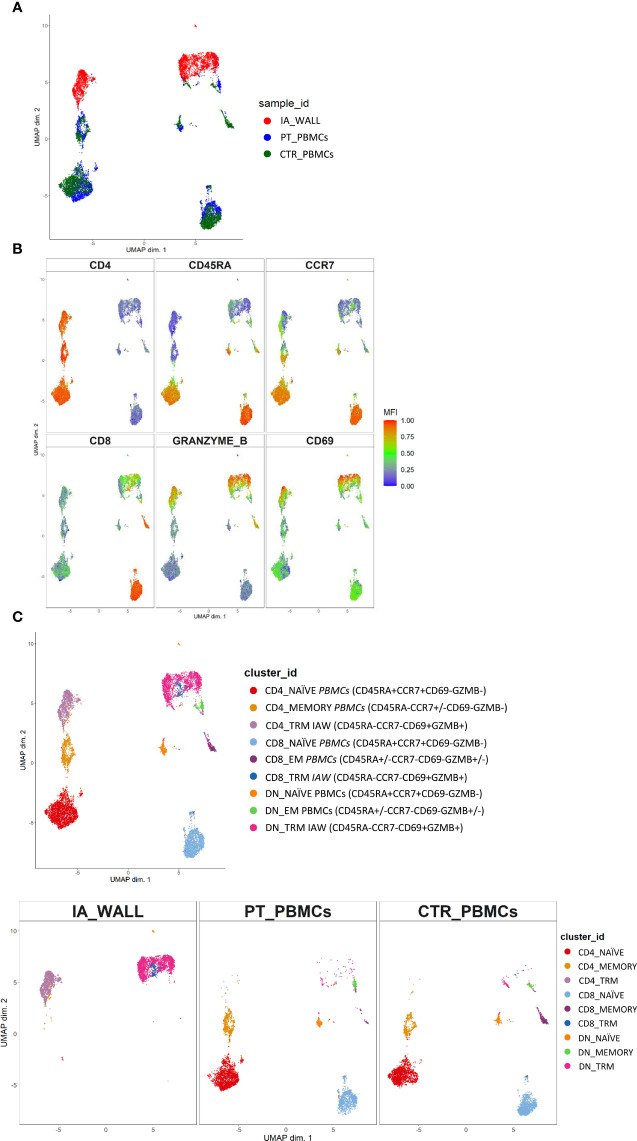
Unsupervised *ex vivo* flow cytometric analysis reveals unique T-cell clusters in the IA wall. **(A)** UMAP projections of normalized expression of markers colored according to the analysed sample. Red: T-cells from the IA wall (IA_WALL), blue: T-cells from peripheral blood of the same patient (PT_PBMCs), green: T-cells from peripheral blood of a pediatric control (CTR_PBMCs). **(B)** Two-dimensional illustration of CD4, CD8, CD45RA, CCR7, GRANZYME B and CD69 expression by UMAP. Blue denotes low, green intermediate and red high expression. **(C)** Nine different T-cell clusters, i.e. three CD4+, three CD8+ and three DN clusters, were identified by UMAP and colored according to cell phenotypes as indicated. CCR7, CD45RA, CD69 and GZMB expression identified three naïve (CCR7+CD45RA+CD69-GZMB-), one memory (CCR7+/-CD45RA-CD69-GZMB-) and two effector memory clusters (“EM”, CCR7-CD45RA+/-CD69-GZMB+/-) in peripheral blood. In addition, three CD69+GZMB+ clusters were identified in the IA wall (“TRM”). Upper panel: overlay of the 3 samples. Lower panels: UMAP Plots stratified according to sample origin: “IA_WALL” (left), “PT_PBMCs” (middle) and “CTR_PBMCs” (right).

T-cells from the IA wall and from peripheral blood were analyzed by multi-dimensional flow cytometry *ex vivo*. UMAP analysis resulted in the clear separation of T-cells derived from the IA wall and T-cells from peripheral blood (**Figure 2A**). UMAPs for CD4 and CD8 expression unveiled that CD4^+^T-cells were separated from CD4^-^T-cells, i. e. from both CD8^+^T-cells and CD4^-^CD8^-^ double-negative (DN) T-cells, in Dimension 1 (**Figure 2B**). Notably, Dimension 2 separated instead T-cells from peripheral blood and from the IA wall, with hardly any overlap between cells from the two sites (**Figures 2A, B**). CD45RA and CCR7 expression unveiled that the major clusters in peripheral blood in the patient and in the pediatric control represented naïve T-cells (**Figure 2B**). Conversely, T-cells from the IA wall expressed high levels of CD69 and GzmB (**Figure 2B**).

We next performed high-resolution, unsupervised clustering and meta-clustering (**Supplementary Figure 1A**) (15). Clustering resolution was chosen based on delta area plots and visual inspection of the expression of 15 markers (**Supplementary Figure 1B**). We first applied an “over-clustering” strategy choosing the maximum number of clusters (K=20) in order to optimally discriminate T cell sub-populations (**Supplementary Figure 1A**). Subsequently, clusters with similar expression profiles (**Supplementary Figure 1B**) were manually annotated. In this way we identified 9 distinguishable major clusters: three CD4+, three CD8+ and three DN T-cell clusters (**Figure 2C** and **Supplementary Figures 2A–C**). Among these three clusters we identified one naïve (CD45RA+CCR7+) and one memory (CD45RA-CCR7+/- in the CD4 compartment) or effector memory subsets (CD45RA+/-CCR7- in the CD4- compartments) that were largely restricted to peripheral blood (**Figure 2C** and **Supplementary Figure 2C**). In addition, we identified in all three T-cell compartments also a third cluster, which was derived from the IA wall and expressed high levels of CD69 and GzmB (**Figure 2C** and **Supplementary Figure 2C**).

Notably, the UMAP analysis suggested that IA T-cells were more closely located to circulating memory T-cells then to naïve T-cells (**Figure 2C**). Indeed, trajectory analysis by DiffusionMap unveiled higher proximity of IA-T cells to circulating memory T-cells as compared to naïve T-cells along the second diffusion component (**Supplementary Figure 3A**). We also observed a clear differentiation pattern from naïve phenotype towards memory in CD4+, CD8+ and DN T-cells (**Supplementary Figure 3B**).

### T-Cells From the IA Wall Lack Canonical Naïve and Memory Subsets, But Display a Phenotype of Cytotoxic Effector Cells

**Figure 3:**
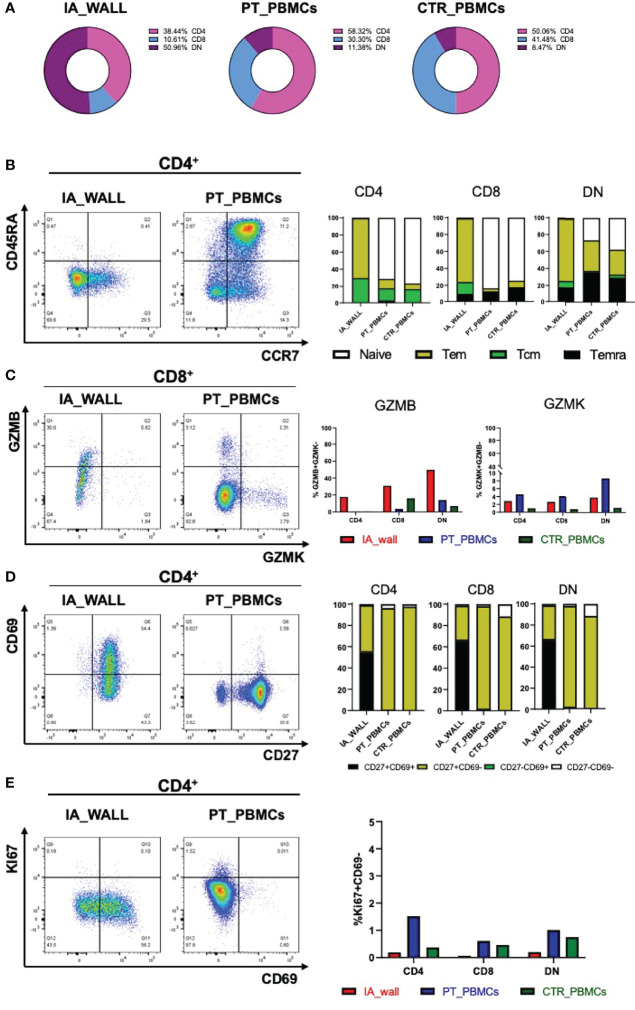
Differentially expressed T-cell differentiation and activation markers in the IA wall. **(A)** Donut plots report frequencies of CD4+ (magenta), CD8+ (violet) and DN cells (blue) among total CD3+T-cells in the IA_WALL and in PBMCs in the three samples. **(B)** CD45RA and CCR7 surface expression was analysed to quantify the presence of naïve T-cells (white) and central/effector memory subsets (colored as indicated) in CD4+ (dot plots and left histogram bars) and in CD8+ and DN T-cells (central and right bar histograms). **(C)** Intracellular GZMB and GZMK expression in CD4+, CD8+ and DN T-cells. Left dot plots show GZMK and GZMB expression in CD8+T-cells in the IA wall and in peripheral blood. Right histogram bars report the frequencies of GZMB/K+ cells among CD4+, CD8+ and DN T-cells in the IA wall (red) and in the blood of the patient (blue) as well as of the pediatric control (green). **(D)** Surface expression of CD69 versus CD27 in CD4+ (dot plots and left bar histograms) as well as in CD8+ and DN T-cells (central and right bar histograms). The frequencies of CD27+/-CD69+/- cells are colored as indicated. **(E)** Intracellular expression of the proliferation marker KI67 versus CD69 surface expression in CD4+T-cells in the IA wall and in peripheral blood from the patient is shown in the left dot plots. Percentage of total KI67+ cells among CD4+, CD8+ and DN T-cells in the IA wall (red), in peripheral blood of the patient (blue) and of the pediatric control (green) is shown in the right panel.

We then compared the T-cell compartments of the IA wall and the blood more in detail. DN T-cells were strongly enriched in the IA wall as compared to peripheral blood, and conventional CD4^+^ and in particular CD8^+^T-cells were consequently reduced (**Figure 3A**, Gating strategy: **Supplementary Figure 4**, absolute cell numbers: **Supplementary Table 2**). We then quantified the co-expression of differentiation-associated surface markers and of intracellular expression of cytotoxic mediators (**Figures 3B–E**). The large majority of CD4^+^T-cells from pediatric blood displayed the canonical CCR7^+^CD45RA^+^ phenotype of naïve T-cells (**Figure 3B**). Similar results were obtained when CD8^+^T-cells from peripheral blood were analyzed, while T-cells with a naive phenotype were virtually absent in the IA wall (**Figure 3B**). CD4^+^T-cells from peripheral blood expressed as expected low levels of GzmB and CD69 (**Figures 3C, D**). In marked contrast, GzmB^+^, but not GzmK^+^, T-cells were overall strongly enriched in the IA wall [**Figure 3C** and **Supplementary Table 3** (reports MFI)]. Moreover, the majority of T-cells from the IA wall expressed the activation/tissue residency marker CD69 (16), but lower levels of the memory-associated costimulatory receptor CD27 (**Figure 3D** and **Supplementary Table 3**). We next analyzed if T-cells from the IA wall were positive for the Ki67 proliferation marker, which is expressed in T-cells that have divided in the last few days. However, both T-cells in peripheral blood and those from the IA wall expressed only very low levels of Ki67 (<2%), indicating that the large majority was in a resting state (**Figure 3E**). This was true for CD4^+^T-cells, CD8^+^T-cells and also for DN T-cells. Notably, also CD69^+^T-cells from the IA wall were largely KI67^-^ (**Figure 3E** and data not shown), consistent with the notion that also in the IA wall CD69 is a marker of tissue-resident rather than of activated cells.

### Composition of the CD4^+^ T-Cell Compartment in the IA Wall

**Figure 4:**
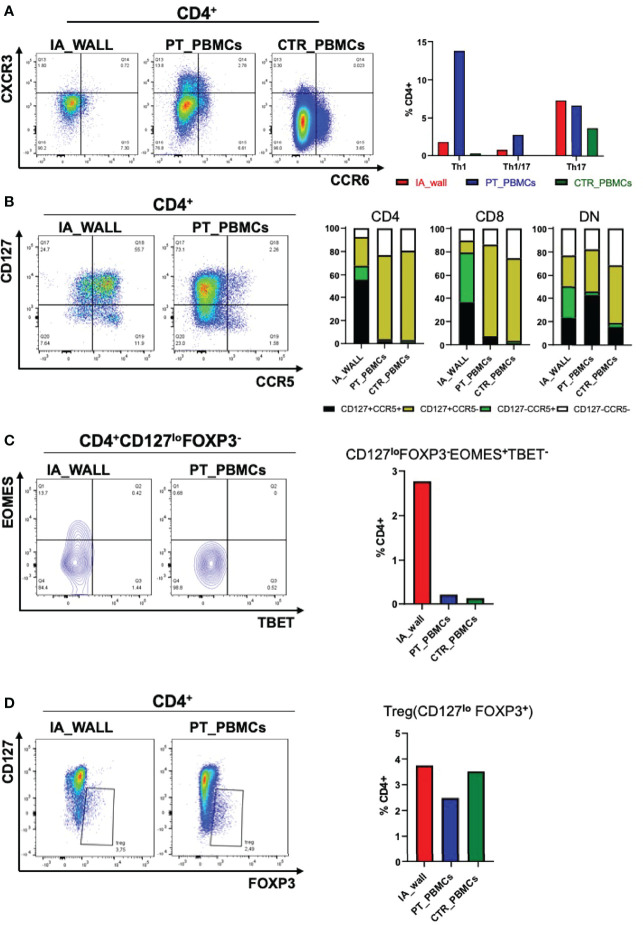
Differential distribution of helper and regulatory T-cell subsets in the IA wall and in peripheral blood. **(A)** Th1 (CXCR3+CCR6-), Th1/17 (CXCR3+ CCR6+) and Th17 (CXCR3- CCR6+) subsets were analysed on gated CD4+ T-cells. Dot plots show CXCR3 versus CCR6 expression in the IA_WALL, in the peripheral blood of the patient (PT_PBMCs, middle) and of the pediatric control (CTR_PBMCs, right). **(B)** CD127 and CCR5 surface expression. Dot plots show CCR5 versus CD127 expression in the IA wall and in the blood of the patient. Bar Histograms report the frequencies of CD127+/-CCR5+/- cells (colored as indicated) among CD4+, CD8+ and in DN T-cells in the 3 samples as indicated. **(C)** EOMES^+^Tr1-like cells were gated first as CD4+CD127^lo^FOXP3- and then as Eomes+T-bet-/lo. Their frequencies among CD4+T-cells was reported in the IA wall (red), in peripheral blood of the patient (blue) and of the pediatric control (green). **(D)** FOXP3+ regulatory T-cells (Treg) cells were gated as CD127^lo^FOXP3+ and their frequencies among CD4+T-cells in the three samples are reported in the right bar histograms as in C.

We next focused on well-defined memory/regulatory subsets of the CD4^+^T-cell compartment (**Figure 4**). An unexpected feature of CD4^+^T-cells in the IA wall was the low expression of CXCR3 and CCR6, well-established differentiation markers of respectively Th1 and Th17 memory cells in the blood (15). In particular, while >15% of cells of the IA patient expressed CXCR3 in peripheral blood, CXCR3^+^ cells were rare in the IA wall (**Figure 4A** and **Supplementary Table 1**). This was also true for CD8^+^T-cells and DN T-cells. Surprisingly, CXCR3^+^ and CCR6^+^CD4^+^T-cells in pediatric blood were highly heterogeneous for CD127 and CCR7 expression, indicating that they contained not only the immature CCR7^+^central memory cells, but also the more differentiated CCR7^-^CD127^+^effector memory (17) and even some CD127^-^effector-like cells (18)(**Supplementary Figure 5A**). In contrast, the few CXCR3^+^ and CCR6^+^CD4^+^T-cells in the IA wall had predominantly an effector memory phenotype. T-cells from pediatric control blood expressed much lower levels of CXCR3 as compared to the IA patient. Importantly, the majority of T-cells from the IA wall expressed instead the chemokine receptor CCR5 (**Figure 4B**), which is known to promote migration to non-lymphoid tissues. This was true both for CD4^+^, CD8^+^ and also DN T-cells. Moreover, a relevant fraction of the CD4^+^CCR5^+^T-cells had down-regulated CD127 expression (**Figure 4B**), a phenotype displayed by Eomes^+^Tr1-like cells (19). Notably, these cells expressed CD27, but lacked CCR6, as is characteristic for these Tr1-like cells (19) (**Supplementary Figure 5B**). Analysis of intracellular transcription factor expression in CD4^+^CD127^lo^FOXP3^-^ cells (gating strategy is shown in **Supplementary Figure 5C**) confirmed that Eomes^+^Tbet^-/lo^ cells were strongly enriched in the IA wall (**Figure 4C**), consistent with the view that these cells represent Eomes^+^Tr1-like cells (19–21). In contrast, FOXP3^+^CD127^lo^Tregs were present at largely similar frequencies in the blood and the IA wall (**Figure 4D**).

### T-Cells From the Aneurism Wall Produce IFN-γ and Contain Poly-Functional Cells

**Figure 5:**
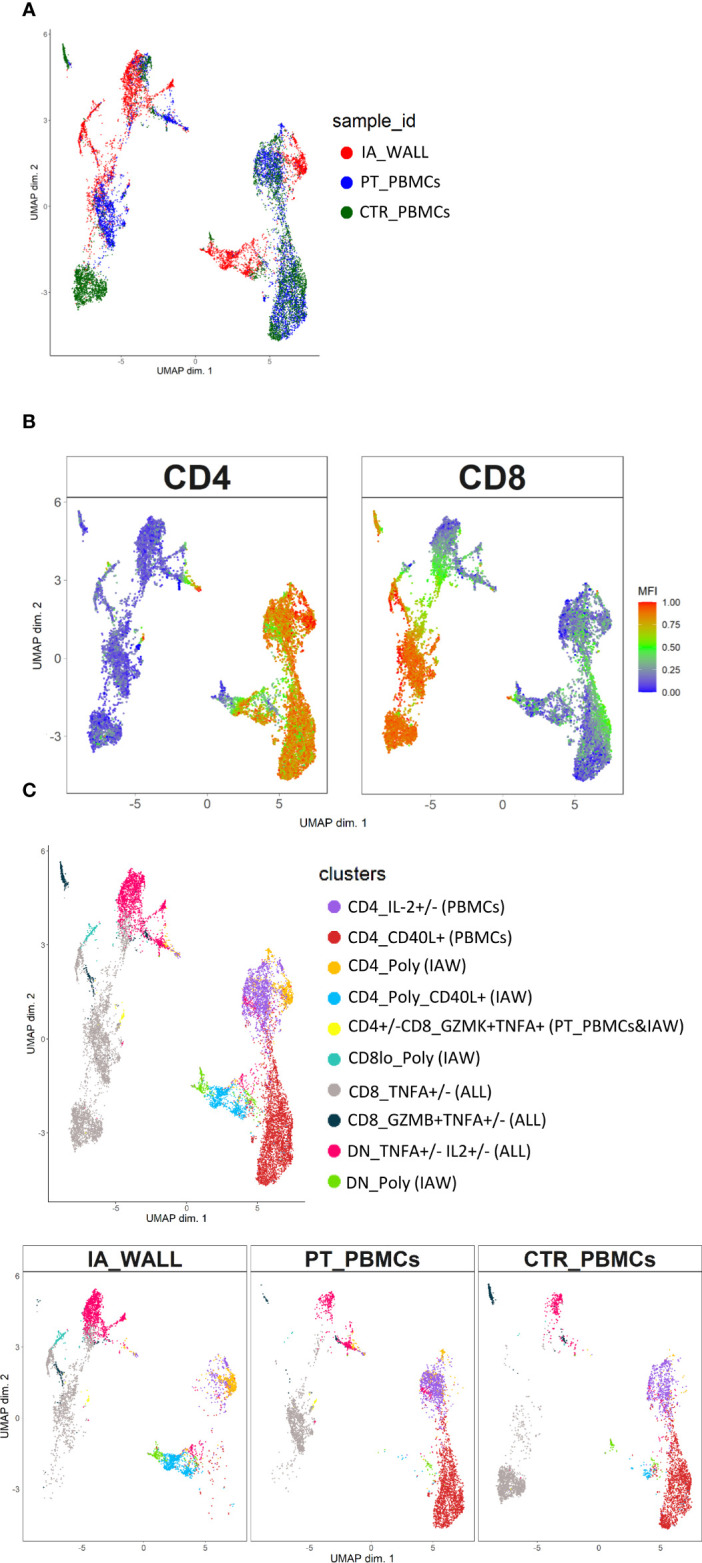
Unsupervised analysis of cytokine producing capacities identifies polyfunctional T-cell clusters in the IA wall. **(A)** UMAP obtained using normalized expression of intracellular cytokines and selected differentiation markers, colored according to the analysed sample (i.e. red: IA_WALL, blue: PT_PBMCs and green: CTR_PBMCs). **(B)** UMAP of CD4 and CD8 expression. Blue denotes low, green intermediate and red high expression. **(C)** UMAP analysis identified ten clusters that were either largely unique for the IA_WALL or for peripheral blood (PBMCs), but also some that were common to all samples (“ALL”). Clusters were colored according to cytokine expression as indicated, the sample of origin is indicated in parenthesis. Clusters that were positive for at least 3 different cytokines were named polyfunctional (“POLY”). The upper UMAP plot shows an overlay of all three samples. The lower UMAP Plots were stratified according to sample of origin: “IA_WALL”, “PT_PBMCs” and “CTR_PBMCs”.

To assess the cytokine producing capacities of T-cells from the IA wall, cells were poly-clonally stimulated and analyzed for intracellular cytokines together with selected differentiation and activation markers (**Supplementary Figures 6A, B**). UMAP analysis unveiled again that T-cells from peripheral blood and the IA wall were largely distinct (**Figure 5A**). It resulted also again in the separation of CD4^+^ from CD4^-^T-cell subsets (**Figure 5B**). We identified 10 different major clusters (**Figure 5C**), including 3 clusters that were abundant in the IA wall, but absent from pediatric blood (**Supplementary Figure 6C**). Intriguingly, these 3 clusters represented CD4+, CD8+ and DN T-cells with polyfunctional capabilities, i.e. that could produce several different cytokines. Conversely, CD4^+^ T-cells that expressed only IL-2 or CD40L were largely restricted to peripheral blood, whereas T-cells that produced TNF-α could be identified both in peripheral blood and in the IA wall (**Figure 5C**).

**Figure 6:**
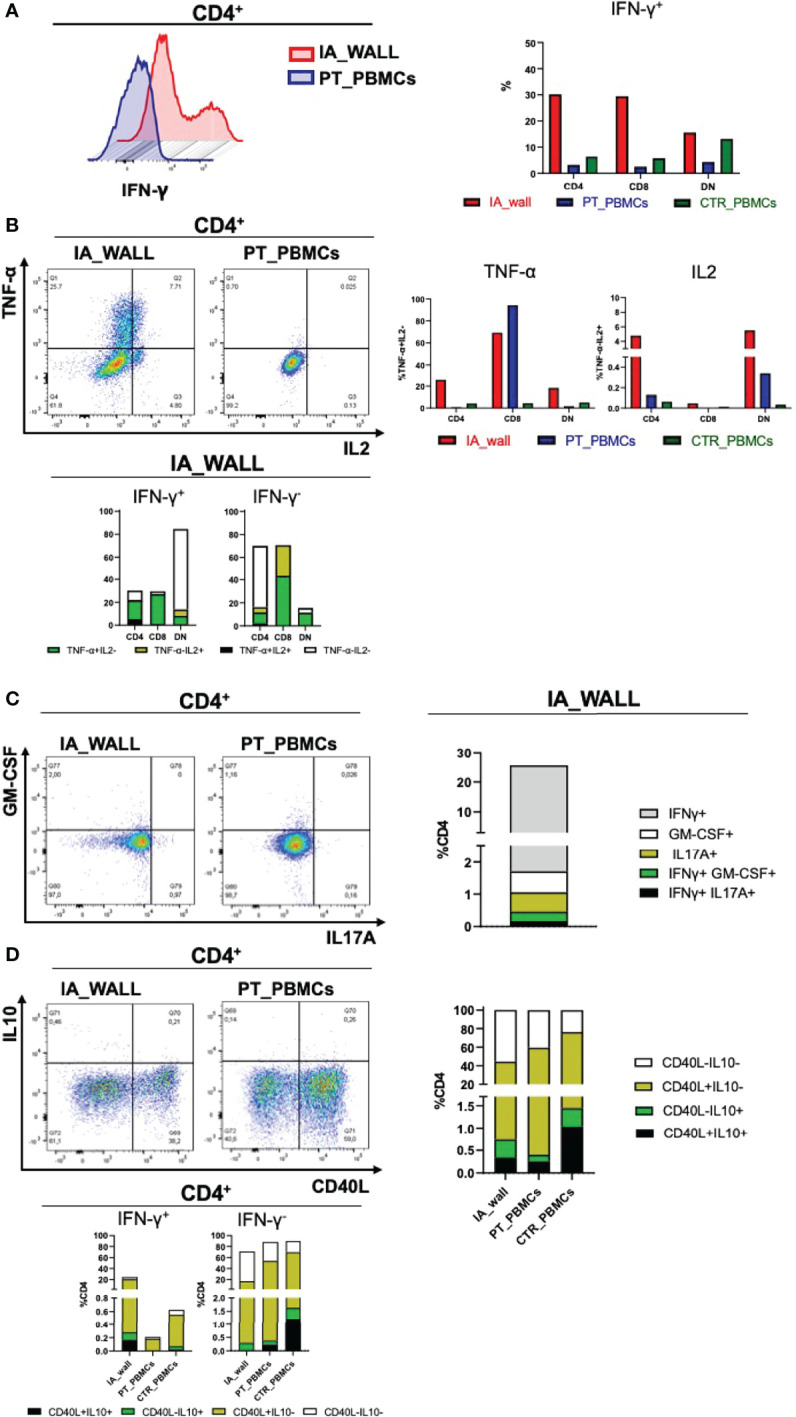
Poly-functional T-cell subsets in the IA wall produce mainly IFN-γ, TNF-α and IL-2 **(A)**. Left histogram overlay shows IFN-γ production by CD4+T-cells in the IA wall and in the blood of the patient. Right panel: Frequencies of IFN-γ producing CD4+, CD8+ and DN T-cells in the IA wall (red) and in peripheral blood of the patient (blue) and of the pediatric control (green). **(B)** Analysis of TNF-α and IL2 production (upper left dot plots and upper right bar histograms). Co-expression of TNF-α, IL2 and IFN-γ in CD4+, CD8+ and DN subsets in the IA wall is shown in the lower panel. Frequencies were calculated on total CD4+, CD8+ and DN cells. TNF-α and IL2 co-expressing cells among IFN-γ+ (right) and IFN-γ - (left) cells are in black, cells that produce neither TNF-α nor IL2 in white, and cells that express only TNF-α or IL2 are colored. **(C)** Co-expression of GM-CSF, IL17A and IFN-γ among CD4+T-cells. Left dot plots show intracellular GM-CSF versus IL17A production in the IA wall and in the blood of the patient. Co-expression with IFN-γ of the two cytokine (IL17 in black and GM-CSF in green) is shown in the right stacked histogram in the IA wall. **(D)** CD40L expression versus IL10 production in CD4+T-cells are show in the left dot plots for CD4+T-cells and in the right stacked histograms in the upper panels for all T-cell subsets. Expression of IL10 and CD40L, alone (coloured as indicated) or in combination (black), in IFN-γ + and in IFN-γ- cells for all samples is shown in the stacked histograms of the lower panels.

A major feature of T-cells in the IA wall was the high production of IFN-γ, TNF and IL-2 from the IA wall (**Figures 6A, B**). Thus, approximately 20% of CD4^+^T-cels from the IA wall produced IFN-γ, while only very few T-cells from peripheral blood were able to do so. This was true for CD4^+^ and CD8^+^ T-cells (**Figure 6A**); only double-negative T-cells from the pediatric control also produced relevant amounts of IFN-γ. T-cells from the IA wall produced also IL-2 and TNF, while in peripheral blood only CD8^+^T-cells from the IA patient produced TNF (**Figure 6B**). Notably, some T-cells from the IA wall were indeed poly-functional, since they co-produced IFN-γ, TNF and IL-2 (**Figure 6B**). CD4^+^T-cells producing IL-17 or GM-CSF were also enriched in the IA wall (**Figure 6C**). They were however rather rare (1-2%), but some of these cells co-produced nevertheless IFN-γ (**Figure 6C**). Finally, CD4^+^T-cells from peripheral blood expressed higher levels of CD40L post-stimulation as compared to CD4^+^T-cells from the IA wall (**Figure 6D**). IL-10 production was overall low (<1-2%), but we detected nevertheless co-expression with IFN-γ and a selective increase of CD4^+^T-cells with an IL-10^+^CD40L^-^ regulatory profile (22) in the IA wall. (**Figure 6D**). In summary, T-cells from the IA wall had overall higher cytokine producing capabilities, and produced in particular high amounts of the Th1-associated cytokines IFN-γ, TNF and IL-2 alone or in combination.

## Discussion

The clinical management of ruptured IA is based on early aneurysm treatment to avoid re-bleeding and ICU admission to prevent and treat secondary brain injuries. Moreover, radiological long-term follow-up are necessary to detect aneurysm recurrence or *de-novo* formation (1). This approach, as in our case report, can lead to favorable outcome even in severe patients. However, the pathogenesis and the natural history of pediatric IAs is still not fully elucidated and investigations aimed at identifying the mechanisms underlying aneurysm rupture could lead to new therapeutic interventions and primary prevention measures.

The role of T-cells in aneurysms is largely unclear (23). A recent study of human abdominal aortic aneurysms unveiled an association of tissue-infiltrating T-cells with disease severity (24). A study of a mouse model for intracranial aneurysms suggested that T-cells could be dispensable for the formation of an aneurysm (10), but their role in aneurysm rupture remained unclear. It seems likely that the role of tissue-infiltrating T-cells in IA depends on their pro- or anti-inflammatory properties. While T-cells were studied in liquor and plasma of patients with ruptured aneurysm and subarachnoid hemorrhage (25, 26), this is to our knowledge the first report that analyses the human T-cell compartment directly in the IA wall by multi-dimensional flow cytometry. We found that the human wall of this pediatric case of a giant IA was heavily infiltrated by T-cells, comprising both CD4^+^ and CD8^+^ as well as CD4^-^CD8^-^ DN T-cells. The latter were enriched, and it seems likely that they contained both α/β and γ/δ-T-cells. Comparison with the T-cell compartment of peripheral blood of the same patient and a pediatric control unveiled that T-cells in the IA wall were completely different from the very well-characterized circulating T-cells, and UMAP analysis confirmed that there was virtually no overlap between the T-cell clusters in the two tissues. Thus, T-cells from pediatric blood contained as expected mainly naïve T-cells, which were in contrast completely absent from the IA wall, excluding also a contamination of the here analyzed tissue sample. A more detailed analysis of antigen-experienced CD4^+^T-cells unveiled further that also conventional CXCR3^+^Th1 and CCR6^+^Th17 memory subsets were large absent in the IA wall. Unexpectedly, however, in spite of the abundance of CXCR3^+^Th1-cells, which included both CCR7^-^ effector memory and CD127^-^ effector-like cells (27), IFN-γ production was hardly detectable in the blood of the SA patient. IFN-γ producing T-cells in the IA wall were in general much more abundant then in the blood, and co-produced several other cytokines, in particular TNF and IL-2, indicating that they were poly-functional. Moreover, they expressed high levels of the cytotoxic effector molecule Granzyme B. Thus, they had a cytotoxic effector phenotype, and this was not only true for CD8^+^ and DN T-cells, but also for CD4^+^T-cells. Notably, T-cells from the IA wall expressed also high levels of the chemokine receptor CCR5, which is considered to be a therapeutic target for recovery after brain injury (28). In addition, they had up-regulated CD69, as is characteristic for tissue-infiltrating T-cells (16). CD69 is rapidly induced upon T-cell activation *in vitro*, but is associated with tissue residency *in vivo*. Indeed, both CD69^+^ and CD69^-^T-cells in the IA wall were largely negative for the proliferation marker Ki67, indicating that they were in a resting state. Finally, regulatory FOXP3^+^CD4^+^T-cells were present at similar low frequencies in peripheral blood and in the IA wall, where they might promote brain repair (29, 30). In addition, Eomes^+^Tr1-like cells (25) were strongly enriched in the IA wall, indicating the presence of different regulatory T-cell subsets. IL-10 is challenging to detect *ex vivo* intracellularly in particular in human tissues (21), and it is thus possible that our analysis underestimated IL-10 production in particular in the IA wall. Nevertheless, we were able to detect an increase of CD4^+^T-cells that expressed IL-10 in the IA wall, consistent with the observed enrichment of Eomes^+^Tr1-like cells that produce very high levels of this anti-inflammatory cytokine. Indeed, IL-10 was produced by CD4^+^T-cells that failed to up-regulate the helper molecule CD40L, as is characteristic for regulatory T-cells (22, 31).

In conclusion, we provided here the first detailed analysis of the T-cell compartment in the IA wall in a pediatric case. The dominance of cytotoxic effector T-cells suggests a predominantly pro-inflammatory, and thus presumably detrimental (32), role of these T-cells in the IA growth and rupture. The strong enrichment of Eomes^+^Tr1-like cells might reflect an unsuccessful attempt to control the large majority of effector cells. Future studies including a larger number of patients with ruptured and unruptured aneurysm could be helpful to understand the role of immunity in this rare and complex disease.

## Data Availability Statement

The original contributions presented in the study are included in the article/**Supplementary Material**. Further inquiries can be directed to the corresponding authors.

## Ethics Statement

The studies involving human participants were reviewed and approved by Ethics Committee of the Fondazione IRCCS Ca’ Granda–Ospedale Maggiore Policlinico. Written informed consent to participate in this study was provided by the participants’ legal guardian/next of kin. Written informed consent was obtained from the minor(s)’ legal guardian/next of kin for the publication of any potentially identifiable images or data included in this article.

## Author Contributions

GM isolated cells, designed and performed flow cytometry analysis and participated in manuscript writing, CV designed and analysed flow cytometry data and participated in Figure's design and manuscript writing, FC and EG performed bioinformatic analysis designed Figures and contributed to manuscript writing, TZ, FO, CM, ERZ and NS provided human specimens and clinical data and partecipated to the study design and manuscript writing ML and MP provided human specimens and clinical data AS pprobided critical discusiion and contributed to manuscript design JG contributed to the study design, coordinated the study and wrote the paper.

## Funding

This study was supported by the Fondazione Cariplo (“Ricerca biomedica condotta da giovani ricercatori” grant number 2019-1632).

## Conflict of Interest

The authors declare that the research was conducted in the absence of any commercial or financial relationships that could be construed as a potential conflict of interest.

## Publisher’s Note

All claims expressed in this article are solely those of the authors and do not necessarily represent those of their affiliated organizations, or those of the publisher, the editors and the reviewers. Any product that may be evaluated in this article, or claim that may be made by its manufacturer, is not guaranteed or endorsed by the publisher.

## Supplementary Material

Supplementary Table 1 List of the used antibodies.

Supplementary Table 2 Absolute cell numbers (CD4+, CD8+ and DN T-cells).

Supplementary Table 3 MFI values of CD127, CCR5, CCR6, GZMB, GZMK, CXCR3, CD27 and CD69 expression among CD4+ T cells.

Supplementary Figure 1 **(A)** UMAP of cells colored according to the clusters as indicated obtained by FlowSOM using K = 20. **(B)** Heatmap of median normalized expression of markers within each cluster. Dendrogram on the left side of the heatmap shows similarities among clusters.

Supplementary Figure 2 **(A)** Histograms of the marker expression values (surface and intracellular differentiation markers analysed *ex vivo*) that were used to distinguish cell clusters. Clusters were colored according to the tissue of origin: IA wall (red), PBMCs (green). **(B)** UMAPs of all analysed markers (as indicated). Blue denotes low, green intermediate and red high expression. **(C)** Percentages of the nine cell clusters.

Supplementary Figure 3 The temporal order of differentiating cells was inferred by DiffusionMap. DiffusionMap, colored by sample of origin **(A)** and clusters **(B)**, show higher proximity of IA wall T-cells to circulating memory T cells as compared to naïve cells. A general differentiation pattern from naïve phenotypes to memory and TRM in CD4+, CD8+ and DN T-cells can be observed.

Supplementary Figure 4 Gating Strategy used to analyse T cell compartment for CD4+, CD8+ and DN T-cell subsets.

Supplementary Figure 5 **(A)** CD127 versus CCR7 expression of gated Th1 (CXCR3+ CCR6-), Th1/17 (CXCR3+ CCR6+) and Th17 (CCR6+) were analysed to identify central memory cells (CCR7+CD127+), effector memory (CCR7- CD127+) and effector-like (CD127- CCR7-) cells. **(B)** To assess the presence of CD27+CCR6- Tr1-like cells the expression of CD27 and CCR6 was analysed on gated CD4+ CD25- CD127- CCR5+ T-cells. **(C)** Gating Strategy used to analyse CD4+ effector-like T-cells (CD127^lo^FOXP3-).

Supplementary Figure 6 **(A)** Histograms of the marker (cytokines and differentiation markers) expression values were used to distinguish cell clusters following brief polyclonal stimulation. Clusters were colored according to the sample of origin: IA_WALL (red), PT_PBMCs and IA_WALL (yellow), PBMCs (blue) and ALL (shared among all tissue, violet). **(B)** UMAP of all analysed markers, as indicated. Blue denotes low, green intermediate and red high expression. **(C)** Frequencies of all cell clusters among T-cells in each sample as indicated.

## References

[1] Krings T Mandell DM Kiehl TR Geibprasert S Tymianski M Alvarez H. Intracranial Aneurysms: From Vessel Wall Pathology to Therapeutic Approach. Nat Rev Neurol (2011) 7:547–59. doi: 10.1038/nrneurol.2011.136 21931350

[2] Beslow LA Jordan LC. Pediatric Stroke: The Importance of Cerebral Arteriopathy and Vascular Malformations. Childs Nerv Syst (2010) 26:1263–73. doi: 10.1007/s00381-010-1208-9 PMC3061823 20625743

[3] Xu R Xie ME Yang W Gailloud P Caplan JM Jackson CM. Epidemiology and Outcomes of Pediatric Intracranial Aneurysms: Comparison With an Adult Population in a 30-Year, Prospective Database. J Neurosurg Pediatr (2021), 28(6):1–10. doi: 10.3171/2021.6.PEDS21268 34507296

[4] Proust F Toussaint P Garnieri J Hannequin D Legars D Houtteville JP. Pediatric Cerebral Aneurysms. J Neurosurg (2001) 94:733–9. doi: 10.3171/jns.2001.94.5.0733 11354404

[5] Aeron G Abruzzo TA Jones BV. Clinical and Imaging Features of Intracranial Arterial Aneurysms in the Pediatric Population. Radiographics (2012) 32:667–81. doi: 10.1148/rg.323105224 22582353

[6] Krings T Geibprasert S terBrugge KG. Pathomechanisms and Treatment of Pediatric Aneurysms. Childs Nerv Syst (2010) 26:1309–18. doi: 10.1007/s00381-009-1054-9 20033187

[7] Etminan N Rinkel GJ. Unruptured Intracranial Aneurysms: Development, Rupture and Preventive Management. Nat Rev Neurol (2016) 12:699–713. doi: 10.1038/nrneurol.2016.150 27808265

[8] Chalouhi N Ali MS Jabbour PM Tjoumakaris SI Gonzalez LF Rosenwasser RH. Biology of Intracranial Aneurysms: Role of Inflammation. J Cereb Blood Flow Metab (2012) 32:1659–76. doi: 10.1038/jcbfm.2012.84 PMC3434628 22781330

[9] Chyatte D Bruno G Desai S Todor DR. Inflammation and Intracranial Aneurysms. Neurosurgery (1999) 45:1137–46; discussion 1146-7. doi: 10.1097/00006123-199911000-00024 10549930

[10] Miyata H Koseki H Takizawa K Kasuya H Nozaki K Narumiya S. T Cell Function is Dispensable for Intracranial Aneurysm Formation and Progression. PloS One (2017) 12:e0175421. doi: 10.1371/journal.pone.0175421 28437485 PMC5402951

[11] Sawyer DM Pace LA Pascale CL Kutchin AC O'Neill BE Starke RM. Lymphocytes Influence Intracranial Aneurysm Formation and Rupture: Role of Extracellular Matrix Remodeling and Phenotypic Modulation of Vascular Smooth Muscle Cells. J Neuroinflamm (2016) 13:185. doi: 10.1186/s12974-016-0654-z PMC4946206 27416931

[12] Frosen J Piippo A Paetau A Kangasniemi M Niemela M Hernesniemi J. Remodeling of Saccular Cerebral Artery Aneurysm Wall is Associated With Rupture: Histological Analysis of 24 Unruptured and 42 Ruptured Cases. Stroke (2004) 35:2287–93. doi: 10.1161/01.STR.0000140636.30204.da 15322297

[13] Muhammad S Chaudhry SR Dobreva G Lawton MT Niemela M Hanggi D. Vascular Macrophages as Therapeutic Targets to Treat Intracranial Aneurysms. Front Immunol (2021) 12:630381. doi: 10.3389/fimmu.2021.630381 33763073 PMC7982735

[14] Filiano AJ Gadani SP Kipnis J. How and Why do T Cells and Their Derived Cytokines Affect the Injured and Healthy Brain? Nat Rev Neurosci (2017) 18:375–84. doi: 10.1038/nrn.2017.39 PMC5823005 28446786

[15] Nowicka M Krieg C Crowell HL Weber LM Hartmann FJ Guglietta S. CyTOF Workflow: Differential Discovery in High-Throughput High-Dimensional Cytometry Datasets. F1000Research (2017) 6:748. doi: 10.12688/f1000research.11622.1 28663787 PMC5473464

[16] Sathaliyawala T Kubota M Yudanin N Turner D Camp P Thome JJ. Distribution and Compartmentalization of Human Circulating and Tissue-Resident Memory T Cell Subsets. Immunity (2013) 38:187–97. doi: 10.1016/j.immuni.2012.09.020 PMC3557604 23260195

[17] Sallusto F Geginat J Lanzavecchia A. Central Memory and Effector Memory T Cell Subsets: Function, Generation, and Maintenance. Annu Rev Immunol (2004) 22:745–63. doi: 10.1146/annurev.immunol.22.012703.104702 15032595

[18] Haringer B Lozza L Steckel B Geginat J. Identification and Characterization of IL-10/IFN-Gamma-Producing Effector-Like T Cells With Regulatory Function in Human Blood. J Exp Med (2009) 206:1009–17. doi: 10.1084/jem.20082238 PMC2715038 19414553

[19] Gruarin P Maglie S De Simone M Haringer B Vasco C Ranzani V. Eomesodermin Controls a Unique Differentiation Program in Human IL-10 and IFN-Gamma Coproducing Regulatory T Cells. Eur J Immunol (2019) 49:96–111. doi: 10.1002/eji.201847722 30431161

[20] Bonnal RJP Rossetti G Lugli E De Simone M Gruarin P Brummelman J. Clonally Expanded EOMES(+) Tr1-Like Cells in Primary and Metastatic Tumors are Associated With Disease Progression. Nat Immunol (2021) 22:735–45. doi: 10.1038/s41590-021-00930-4 34017124

[21] Cossarizza A Chang HD Radbruch A Abrignani S Addo R Akdis M. Guidelines for the Use of Flow Cytometry and Cell Sorting in Immunological Studies (Third Edition). Eur J Immunol (2021) 51:2708–3145. 34910301 10.1002/eji.202170126 PMC11115438

[22] Facciotti F Gagliani N Haringer B Alfen JS Penatti A Maglie S. IL-10-Producing Forkhead Box Protein 3-Negative Regulatory T Cells Inhibit B-Cell Responses and are Involved in Systemic Lupus Erythematosus. J Allergy Clin Immunol (2016) 137:318–21.e5. doi: 10.1016/j.jaci.2015.06.044 26318071

[23] Lv BJ Li J Cheng X. T Lymphocytes and Aortic Aneurysms. Sci China Life Sci (2014) 57:795–801. doi: 10.1007/s11427-014-4699-x 25104452

[24] Sagan A Mikolajczyk TP Mrowiecki W MacRitchie N Daly K Meldrum A. T Cells Are Dominant Population in Human Abdominal Aortic Aneurysms and Their Infiltration in the Perivascular Tissue Correlates With Disease Severity. Front Immunol (2019) 10:1979. doi: 10.3389/fimmu.2019.01979 31552015 PMC6736986

[25] Mohme M Sauvigny T Mader MM Schweingruber N Maire CL Runger A. Immune Characterization in Aneurysmal Subarachnoid Hemorrhage Reveals Distinct Monocytic Activation and Chemokine Patterns. Transl Stroke Res (2020) 11:1348–61. doi: 10.1007/s12975-019-00764-1 31858408

[26] Roa JA Sarkar D Zanaty M Ishii D Lu Y Karandikar NJ. Preliminary Results in the Analysis of the Immune Response After Aneurysmal Subarachnoid Hemorrhage. Sci Rep (2020) 10:11809. doi: 10.1038/s41598-020-68861-y 32678268 PMC7367262

[27] Geginat J Paroni M Facciotti F Gruarin P Kastirr I Caprioli F. The CD4-Centered Universe of Human T Cell Subsets. Semin Immunol (2013) 25:252–62. doi: 10.1016/j.smim.2013.10.012 24183700

[28] Joy MT Ben Assayag E Shabashov-Stone D Liraz-Zaltsman S Mazzitelli J Arenas M. CCR5 Is a Therapeutic Target for Recovery After Stroke and Traumatic Brain Injury. Cell (2019) 176:1143–57.e13. 30794775 10.1016/j.cell.2019.01.044 PMC7259116

[29] Shi L Sun Z Su W Xu F Xie D Zhang Q. Treg Cell-Derived Osteopontin Promotes Microglia-Mediated White Matter Repair After Ischemic Stroke. Immunity (2021) 54:1527–42.e8. doi: 10.1016/j.immuni.2021.04.022 34015256 PMC8282725

[30] Ito M Komai K Mise-Omata S Iizuka-Koga M Noguchi Y Kondo T. Brain Regulatory T Cells Suppress Astrogliosis and Potentiate Neurological Recovery. Nature (2019) 565:246–50. doi: 10.1038/s41586-018-0824-5 30602786

[31] Geginat J Vasco M Gerosa M Tas SW Pagani M Grassi F. IL-10 Producing Regulatory and Helper T-Cells in Systemic Lupus Erythematosus. Semin Immunol (2019) 44:101330. doi: 10.1016/j.smim.2019.101330 31735515

[32] Iadecola C Anrather J. The Immunology of Stroke: From Mechanisms to Translation. Nat Med (2011) 17:796–808. doi: 10.1038/nm.2399 21738161 PMC3137275

