# Supplementary material for: Deep Phenotyping of T-Cells Derived From the Aneurysm Wall in a Pediatric Case of Subarachnoid Hemorrhage
Source: Front Immunol. 2022 May 31;13:866558. doi: 10.3389/fimmu.2022.866558 (PMC9197186; doi:10.3389/fimmu.2022.866558)
Supplement: Supplementary Table 1 — List of the used antibodies. [file Table_1.docx]

| **Marker** | **Clone** | **Catalog Num** | **Brand** |
| --- | --- | --- | --- |
| BUV563 Mouse Anti-Human CD4 | SK3 | 612912 | BD Horizon™ |
| BUV737 Mouse Anti-Human CD3 | UCHT1 | 612750 | BD Horizon™ |
| BUV805 Mouse Anti-Human CD8 | SK1 | 612889 | BD Horizon™ |
| BB700 Mouse Anti-Human CD127 | HIL-7R-M21 | 566398 | BD Horizon™ |
| APC-R700 Mouse Anti-Human CD25 | 2A3 | 565106 | BD Horizon™ |
| BV480 Mouse Anti-Human CD27 | L128 | 566139 | BD Horizon™ |
| BV786 Mouse Anti-Human CD69 | FN50 | 563834 | BD Horizon™ |
| BV605 Mouse Anti-Human CD45RA | HI100 | 562886 | BD Horizon™ |
| BUV496 Mouse Anti-Human CD196 (CCR6) | 11A9 | 612948 | BD Horizon™ |
| BUV395 Mouse Anti-Human CD195(CCR5) | 2D7/CCR5 | 565224 | BD Horizon™ |
| PE-CF594 Mouse Anti-Human CD197 (CCR7) | 150503 | 562381 | BD Horizon™ |
| PE-Cy™7 Mouse Anti-Human CD183 (CXCR3) | 1C6/CXCR3 | 560831 | BD Pharmingen™ |
| PE/Cyanine5 anti-human CD154 (CD40L) | 24-31 | 310808 | Biolegend |
| Brilliant Violet 650™ anti-human CD137 (4-1BB) | 4B4-1 | 309828 | Biolegend |
| Alexa Fluor® 488 Mouse anti-Human FoxP3 | 259D/C7 | 560047 | BD Pharmingen™ |
| PE granzyme K Monoclonal antibody | GM6C3 | sc-56125 | Santa Cruz Biotechnology, INC |
| eFluor 660 EOMES Monoclonal Antibody | WD1928 | 50-4877-41 | eBioscience™ |
| BV421 Mouse Anti-Human Granzyme B | GB11 | 563389 | BD Horizon™ |
| BV711 Mouse Anti-Ki-67 | B56 | 563755 | BD Horizon™ |
| BV750 Mouse Anti-Human IFN-γ | B27 | 566357 | BD Horizon™ |
| PE-CF594 Mouse Anti-T-bet | O4-46 | 562467 | BD Horizon™ |
| APC-R700 Mouse Anti-Human IL-17A | N49-653 | 565163 | BD Horizon™ |
| BV650 Rat Anti-Human and Viral IL-10 | JES3-9D7 | 564051 | BD Horizon™ |
