# Supplementary material for: Deep Phenotyping of T-Cells Derived From the Aneurysm Wall in a Pediatric Case of Subarachnoid Hemorrhage
Source: Front Immunol. 2022 May 31;13:866558. doi: 10.3389/fimmu.2022.866558 (PMC9197186; doi:10.3389/fimmu.2022.866558)
Supplement: Supplementary Table 2 — Absolute cell numbers (CD4+, CD8+ and DN T-cells). [file Table_2.docx]

| # Cells | CD4 | CD8 | DN |
| --- | --- | --- | --- |
| IA WALL | 12009 | 3316 | 15945 |
| PT_PBMC | 44346 | 23088 | 8646 |
| PB Control | 293688 | 243557 | 49719 |
