# Supplementary figures and images for: Deep Phenotyping of T-Cells Derived From the Aneurysm Wall in a Pediatric Case of Subarachnoid Hemorrhage
Source: Front Immunol. 2022 May 31;13:866558. doi: 10.3389/fimmu.2022.866558 (PMC9197186; doi:10.3389/fimmu.2022.866558)

A)

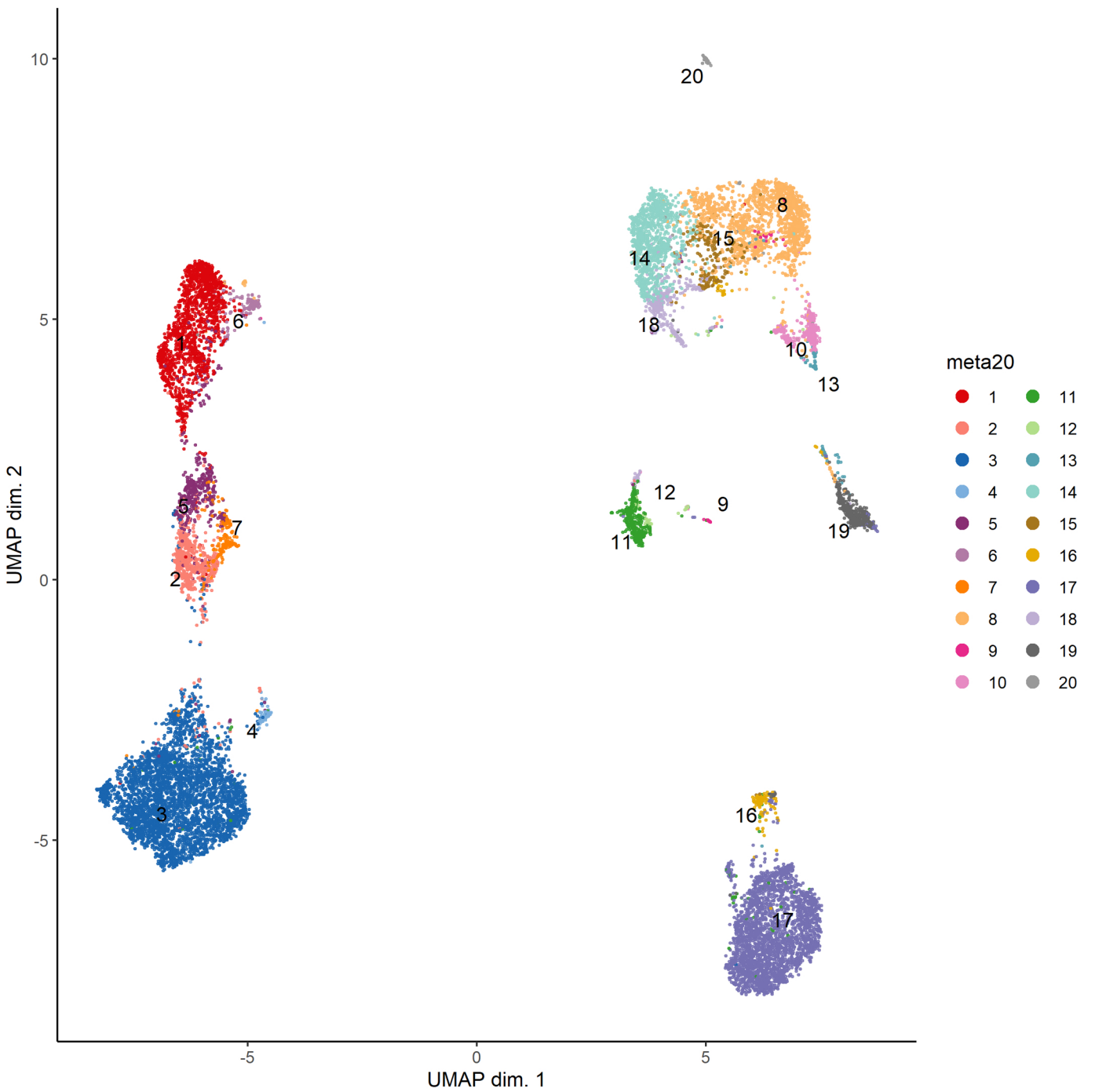

B)

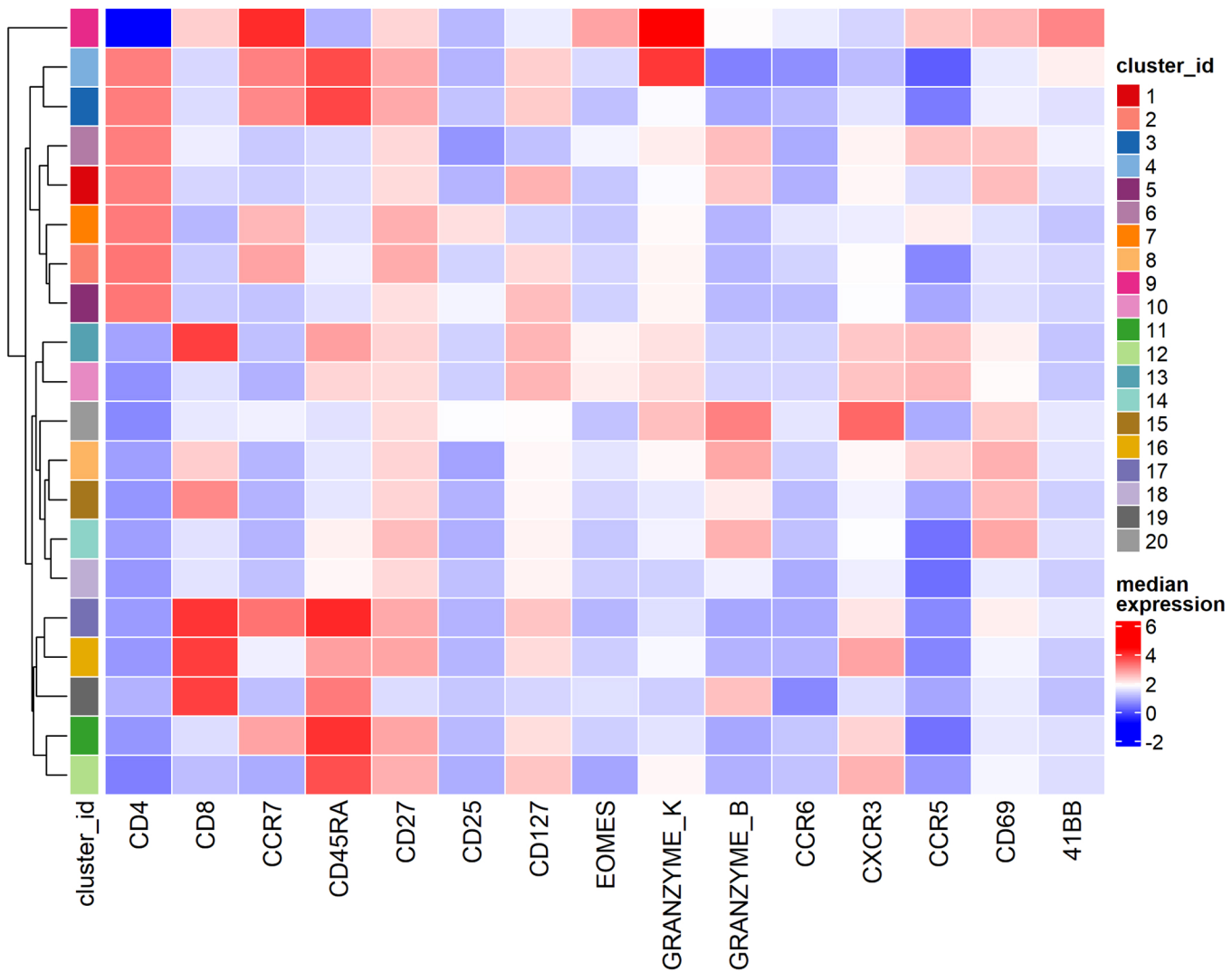

Supplementary figure 1

Supplement: Supplementary Figure 1 — (A) UMAP of cells colored according to the clusters as indicated obtained by FlowSOM using K = 20. (B) Heatmap of median normalized expression of markers within each cluster. Dendrogram on the left side of the heatmap shows similarities among clusters. [file Image_1.pdf]

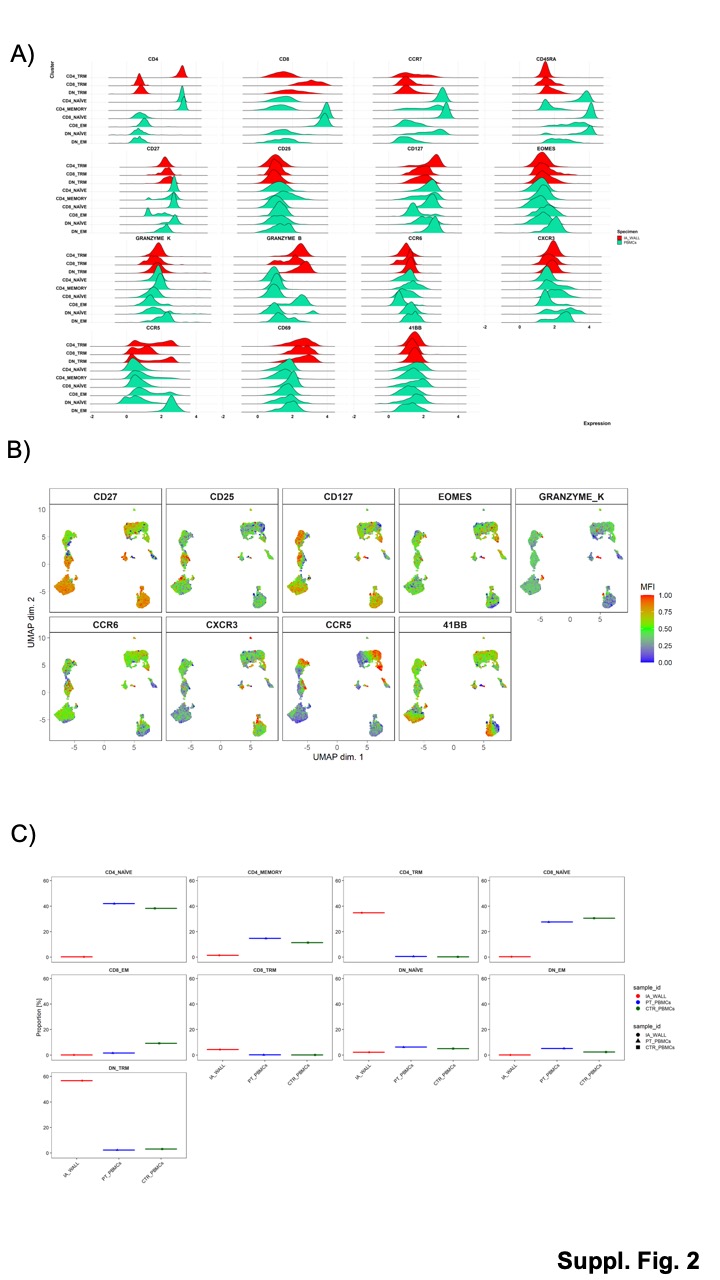

Supplement: Supplementary Figure 2 — (A) Histograms of the marker expression values (surface and intracellular differentiation markers analysed ex vivo) that were used to distinguish cell clusters. Clusters were colored according to the tissue of origin: IA wall (red), PBMCs (green). (B) UMAPs of all analysed markers (as indicated). Blue denotes low, green intermediate and red high expression. (C) Percentages of the nine cell clusters. [file Image_2.jpeg]

A)

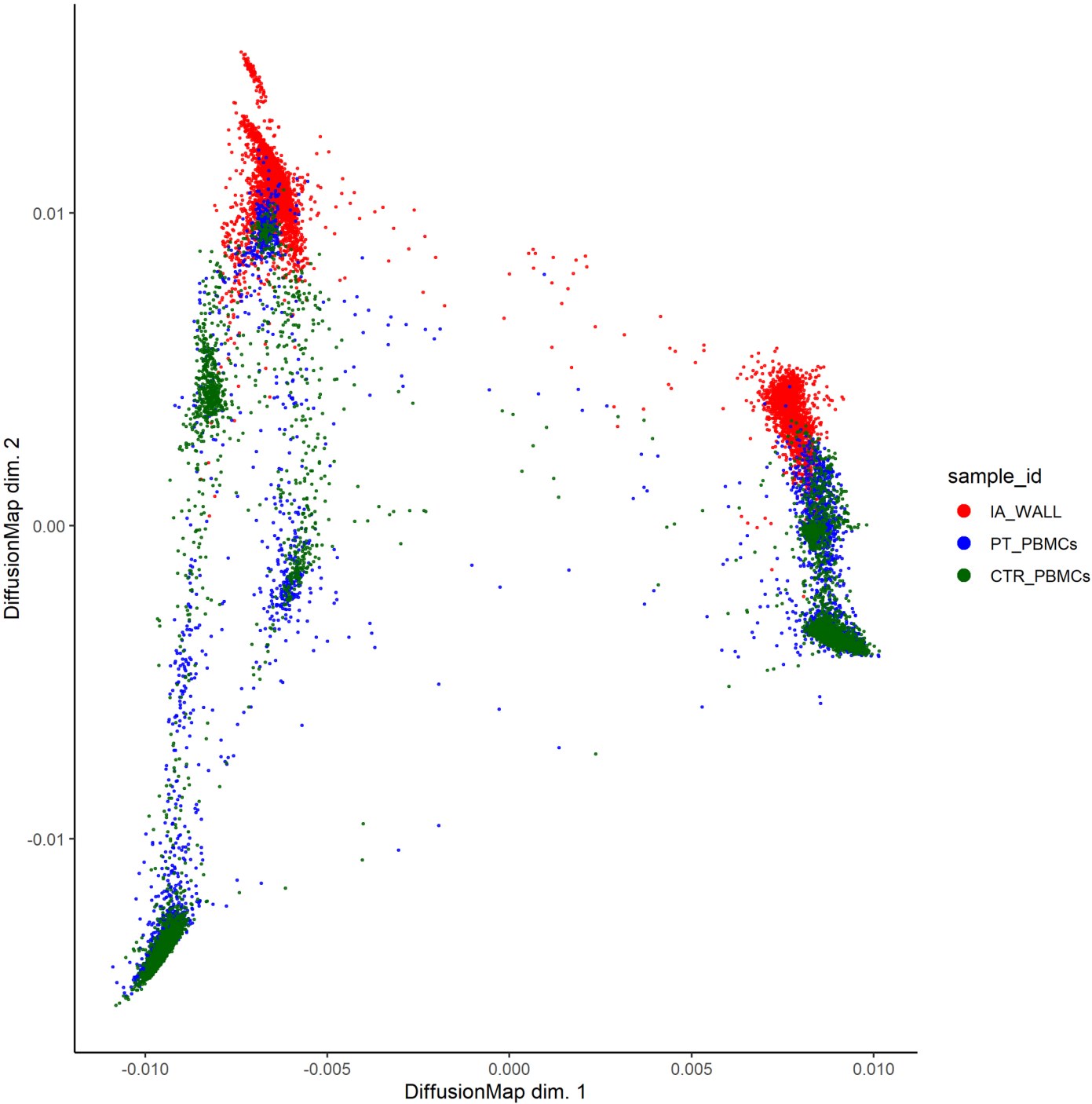

B)

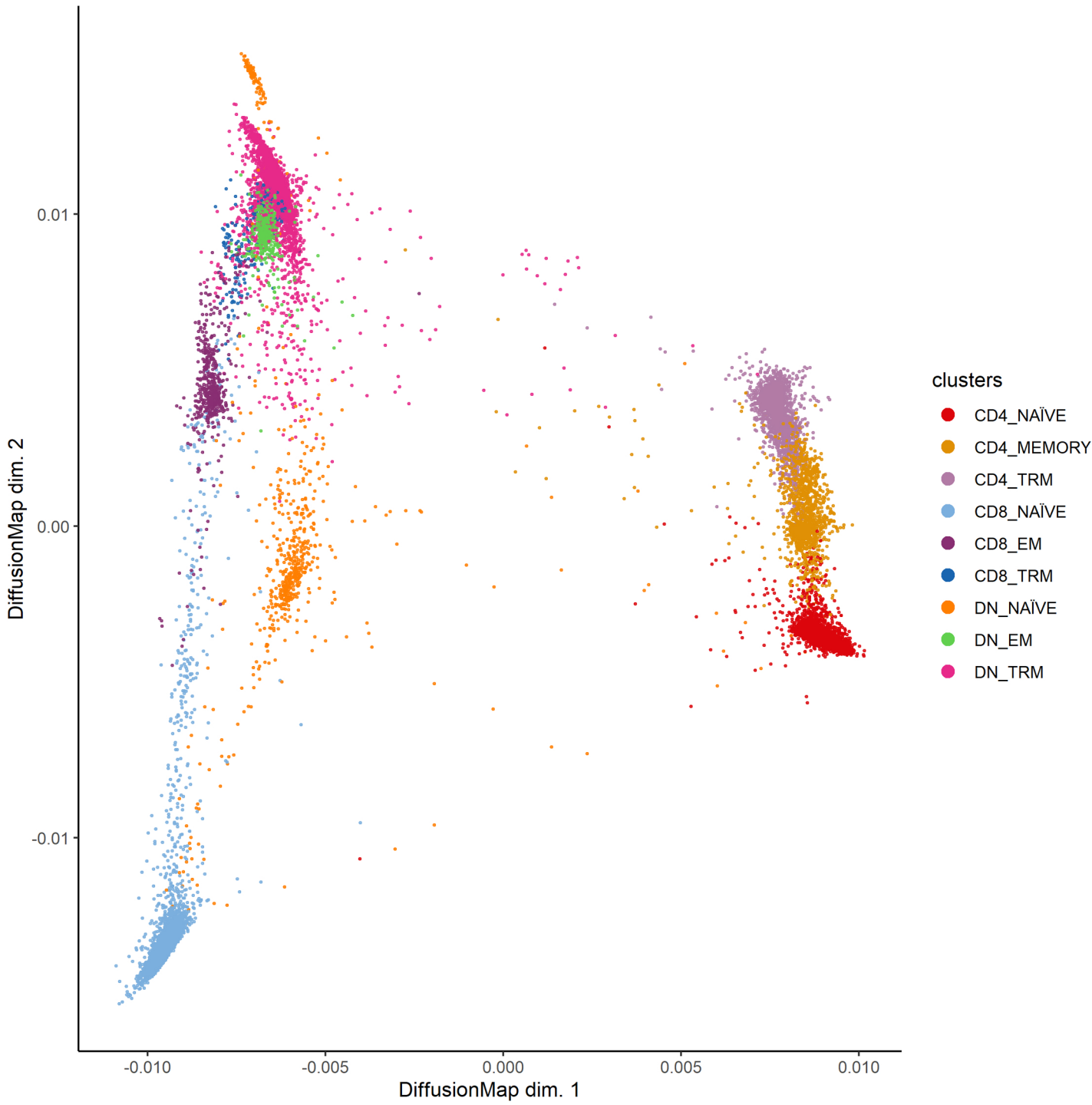

Supplementary figure 3

Supplement: Supplementary Figure 3 — The temporal order of differentiating cells was inferred by DiffusionMap. DiffusionMap, colored by sample of origin (A) and clusters (B), show higher proximity of IA wall T-cells to circulating memory T cells as compared to naïve cells. A general differentiation pattern from naïve phenotypes to memory and TRM in CD4+, CD8+ and DN T-cells can be observed. [file Image_3.pdf]

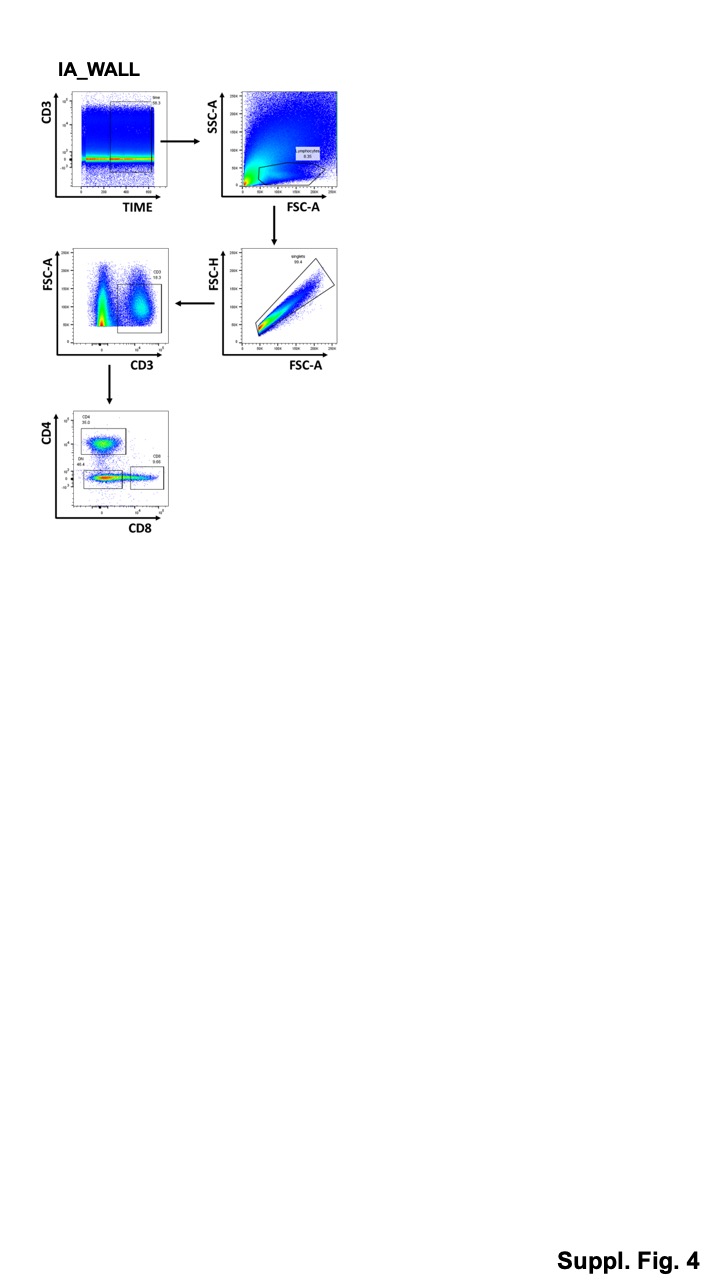

Supplement: Supplementary Figure 4 — Gating Strategy used to analyse T cell compartment for CD4+, CD8+ and DN T-cell subsets. [file Image_4.jpeg]

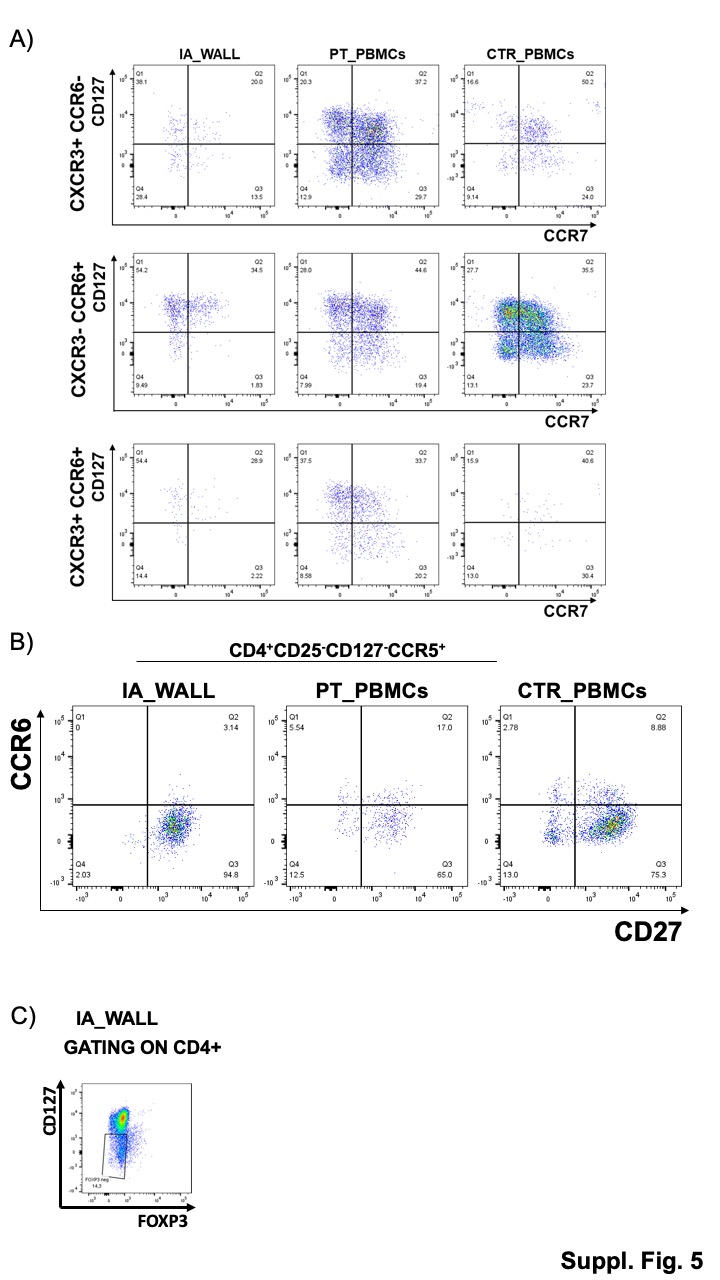

Supplement: Supplementary Figure 5 — (A) CD127 versus CCR7 expression of gated Th1 (CXCR3+ CCR6-), Th1/17 (CXCR3+ CCR6+) and Th17 (CCR6+) were analysed to identify central memory cells (CCR7+CD127+), effector memory (CCR7- CD127+) and effector-like (CD127- CCR7-) cells. (B) To assess the presence of CD27+CCR6- Tr1-like cells the expression of CD27 and CCR6 was analysed on gated CD4+ CD25- CD127- CCR5+ T-cells. (C) Gating Strategy used to analyse CD4+ effector-like T-cells (CD127loFOXP3-). [file Image_5.jpeg]

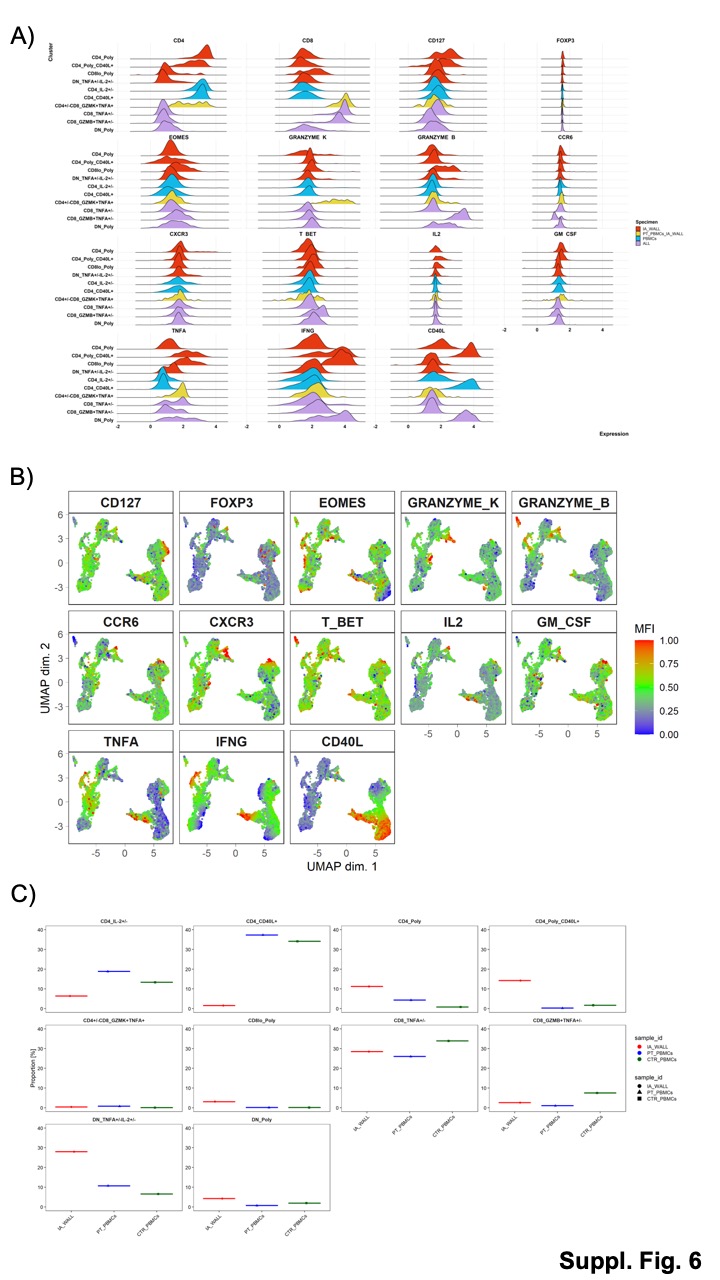

Supplement: Supplementary Figure 6 — (A) Histograms of the marker (cytokines and differentiation markers) expression values were used to distinguish cell clusters following brief polyclonal stimulation. Clusters were colored according to the sample of origin: IA_WALL (red), PT_PBMCs and IA_WALL (yellow), PBMCs (blue) and ALL (shared among all tissue, violet). (B) UMAP of all analysed markers, as indicated. Blue denotes low, green intermediate and red high expression. (C) Frequencies of all cell clusters among T-cells in each sample as indicated. [file Image_6.jpeg]
